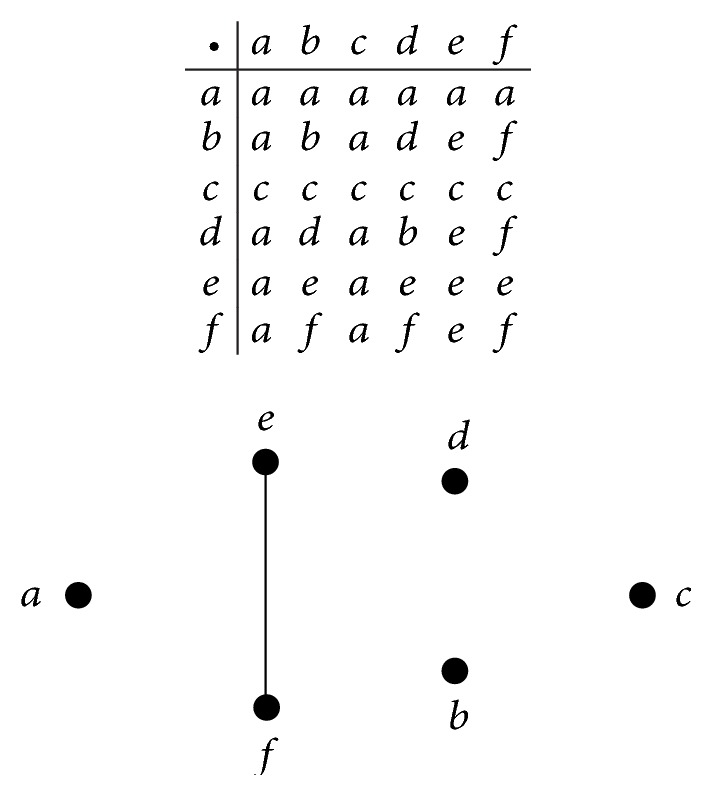# Concave Soft Sets, Critical Soft Points, and Union-Soft Ideals of Ordered Semigroups

**DOI:** 10.1155/2014/467968

**Published:** 2014-10-19

**Authors:** Young Bae Jun, Seok Zun Song, G. Muhiuddin

**Affiliations:** ^1^Department of Mathematics Education, Gyeongsang National University, Jinju 660-701, Republic of Korea; ^2^Department of Mathematics, Jeju National University, Jeju 690-756, Republic of Korea; ^3^Department of Mathematics, University of Tabuk, P.O. Box 741, Tabuk 71491, Saudi Arabia

## Abstract

The notions of union-soft semigroups, union-soft *l*-ideals, and union-soft *r*-ideals are introduced, and related properties are investigated. Characterizations of a union-soft semigroup, a union-soft *l*-ideal, and a union-soft *r*-ideal are provided. The concepts of union-soft products and union-soft semiprime soft sets are introduced, and their properties related to union-soft *l*-ideals and union-soft *r*-ideals are investigated. Using the notions of union-soft *l*-ideals and union-soft *r*-ideals, conditions for an ordered semigroup to be regular are considered. The concepts of concave soft sets and critical soft points are introduced, and their properties are discussed.

## 1. Introduction

The uncertainty which has appeared in economics, engineering, environmental science, medical science and social science, and so forth is too complicated to be captured within a traditional mathematical framework. Molodtsov's soft set theory [1] is a kind of new mathematical model for coping with uncertainty from a parameterization point of view. In soft set theory, the problem of setting the membership function does not arise, which makes the theory easily applied to many different fields. At present, works on the soft set theory with algebraic applications are progressing rapidly (see [2–5]). Mainly, Kehayopulu et al. studied ordered semigroups (see [6–10]). Feng et al. discussed soft relations in semigroups (see [11]) and explored decomposition of fuzzy soft sets with finite value spaces (see [12]). Also, Feng and Li [13] considered soft product operations. Jun et al. [14] applied the concept of soft set theory to ordered semigroups. They applied the notion of soft sets by Molodtsov to ordered semigroups and introduced the notions of (trivial, whole) soft ordered semigroups, soft ordered subsemigroups, soft *l*-ideals, soft *r*-ideals, and *l*-idealistic and *r*-idealistic soft ordered semigroups. They investigated various related properties.

The aim of this paper is to lay a foundation for providing a soft algebraic tool (in ordered semigroups) in considering many problems that contain uncertainties. We introduce the notions of union-soft semigroups, union-soft *l*-ideals, and union-soft *r*-ideals and investigate their properties. We consider characterizations of a union-soft semigroup, a union-soft *l*-ideal, and a union-soft *r*-ideal. We introduce the concepts of union-soft products and union-soft semiprime soft sets and investigate their properties related to union-soft *l*-ideals and union-soft *r*-ideals. Using the notions of union-soft *l*-ideals and union-soft *r*-ideals, we provide conditions for an ordered semigroup to be regular. We also introduce the concepts of concave soft sets and critical soft points and discuss their properties.

## 2. Preliminaries

### 2.1. Basic Results on Ordered Semigroups

An* ordered semigroup* (or,* po-semigroup*) is an ordered set (*S*, ≤) which is a semigroup such that

(1) $$\begin{array}{c} \left(\forall a,b,x\in S\right)\;\left(a\leq b⟹xa\leq xb,\;\;ax\leq bx\right). \end{array}$$

For *A*⊆*S*, we denote

(2) $$\begin{array}{c} \left(A\right]:=\left\{t\in S\;|\;t\leq h\;\;\text{for}\;\;\text{some}\;\;h\in A\right\}. \end{array}$$

For any *a* ∈ *S*, denote by *R*(*a*) (resp., *L*(*a*)) the right (resp., left) ideal of *S* generated by *a*. Note that *R*(*a*) = (*a* ∪ *aS*] and *L*(*a*) = (*a* ∪ *Sa*].

An ordered semigroup *S* is said to be

(i) *left* (resp.,* right*)* regular* if it satisfies
  (3) $$\begin{array}{c} \left(\forall a\in S\right)\;\left(\exists x\in S\right)\;\left(a\leq xa^{2}\right)\;\left(\text{resp}.\;\;a\leq a^{2}x\right); \end{array}$$
(ii) *regular* if it satisfies
  (4) $$\begin{array}{c} \left(\forall a\in S\right)\;\left(\exists x\in S\right)\;\left(a\leq axa\right); \end{array}$$
(iii) *intraregular* if it satisfies
  (5) $$\begin{array}{c} \left(\forall a\in S\right)\;\left(\exists x,y\in S\right)\;\left(a\leq xa^{2}y\right). \end{array}$$

**Lemma 1 (see [9]).**

An ordered semigroup *S* is regular if and only if

(6) $$\begin{array}{c} \left(\forall a\in S\right)\left(R\left(a\right)\cap L\left(a\right)\subseteq \left(R\left(a\right)L\left(a\right)\right]\right). \end{array}$$

A nonempty subset *I* of an ordered semigroup *S* is called a* left* (resp.,* right*)* ideal* of *S* if

(7) $$\begin{array}{c} SI\subseteq I\;\left(\text{resp}.\;\;IS\subseteq I\right).\\ \left(\forall a,b\in S\right)\;\left(a\in I,\;\;b\leq a⟹b\in I\right). \end{array}$$

### 2.2. Basic Results on Soft Sets

A soft set theory is introduced by Molodtsov [1], and Çağman and Enginoğlu [15] provided new definitions and various results on soft set theory.

In what follows, let *U* be an initial universe set and *E* be a set of parameters. Let *P*(*U*) denotes the power set of *U* and *I*, *J*,…⊆*E*.

**Definition 2 (see [1, 15]).**

A soft set (*F*, *I*) over *U* is defined to be the set of ordered pairs

(8) $$\begin{array}{c} \left(F,I\right):=\left\{\left(x,F\left(x\right)\right):x\in E,\;F\left(x\right)\in P\left(U\right)\right\}, \end{array}$$

where *F* : *E* → *P*(*U*) such that *F*(*x*) = *∅* if *x* ∉ *I*.

For a soft set (*F*, *I*) over *U* and a subset *δ* of *U*, the *δ-exclusive set* of (*F*, *I*), denoted by *e*_*I*_(*F*; *δ*), is defined to be the set

(9) $$\begin{array}{c} e_{I}\left(F;\delta \right):=\left\{x\in I\;|\;\delta ⊇F\left(x\right)\right\}. \end{array}$$

For any soft sets (*F*, *E*) and (*G*, *E*) over *U*, we define

(10) $$\begin{array}{c} \left(F,E\right)\tilde{\subseteq }\left(G,E\right)\;\;\text{if}\;\;F\left(x\right)\subseteq G\left(x\right)\;\forall x\in E. \end{array}$$

The* soft union*, denoted by $\left(F,E)\;\tilde{\cup }\;(G,E\right)$, of (*F*, *E*) and (*G*, *E*) is defined to be the soft set $\left(F\;\tilde{\cup }\;G,E\right)$ over *U* in which $F\;\tilde{\cup }\;G$ is defined by

(11) $$\begin{array}{c} \left(F\;\tilde{\cup }\;G\right)\left(x\right)=F\left(x\right)\cup G\left(x\right)\;\forall x\in E. \end{array}$$

The* soft intersection*, denoted by $\left(F,E)\;\tilde{\cap }\;(G,E\right)$, of (*F*, *E*) and (*G*, *E*) is defined to be the soft set $\left(F\;\tilde{\cap }\;G,E\right)$ over *U* in which $F\;\tilde{\cap }\;G$ is defined by

(12) $$\begin{array}{c} \left(F\;\tilde{\cap }\;G\right)\left(x\right)=F\left(x\right)\cap G\left(x\right)\;\forall x\in E. \end{array}$$

## 3. Union-Soft Ideals

In what follows, we take *E* = *S* as a set of parameters, which is an ordered semigroup unless otherwise specified.

**Definition 3 .**

A soft set (*F*, *S*) over *U* is called a union-soft semigroup over *U* if it satisfies

(13) $$\begin{array}{c} \left(\forall x,y\in S\right)\;\left(F\left(xy\right)\subseteq F\left(x\right)\cup F\left(y\right)\right). \end{array}$$

**Theorem 4 .**

A soft set (*F*, *S*) over *U* is a union-soft semigroup over *U* if and only if the nonempty *δ*-exclusive set of (*F*, *S*) is a subsemigroup of *S* for all *δ*⊆*U*.

**Proof**

Assume that (*F*, *S*) over *U* is a union-soft semigroup over *U*. Let *δ*⊆*U* be such that *e*_*S*_(*F*; *δ*) ≠ *∅*. Let *x*, *y* ∈ *e*_*S*_(*F*; *δ*). Then *F*(*x*)⊆*δ* and *F*(*y*)⊆*δ*. It follows from (13) that

(14) $$\begin{array}{c} F\left(xy\right)\subseteq F\left(x\right)\cup F\left(y\right)\subseteq \delta , \end{array}$$

so that *xy* ∈ *e*_*S*_(*F*; *δ*). Thus *e*_*S*_(*F*; *δ*) is a subsemigroup of *S*.

Conversely, suppose that the nonempty *δ*-exclusive set of (*F*, *S*) is a subsemigroup of *S* for all *δ*⊆*U*. Let *x*, *y* ∈ *S* be such that *F*(*x*) = *δ*_*x*_ and *F*(*y*) = *δ*_*y*_. Taking *δ* = *δ*_*x*_ ∪ *δ*_*y*_ implies that *x*, *y* ∈ *e*_*S*_(*F*; *δ*). Hence *xy* ∈ *e*_*S*_(*F*; *δ*), and so *F*(*xy*)⊆*δ* = *δ*_*x*_ ∪ *δ*_*y*_ = *F*(*x*) ∪ *F*(*y*). Therefore (*F*, *S*) is a union-soft semigroup over *U*.

**Definition 5 .**

For a left ideal *A* of *S*, a soft set (*F*, *A*) over *U* is called a* union-soft l-ideal* over *U* related to *A* if it satisfies

(15) $$\begin{array}{c} \left(\forall x\in S\right)\;\left(\forall a\in A\right)\;\left(F\left(xa\right)\subseteq F\left(a\right)\right), \end{array}$$

(16) $$\begin{array}{c} \left(\forall x,y\in A\right)\;\left(x\leq y\Rightarrow F\left(x\right)\subseteq F\left(y\right)\right). \end{array}$$

**Definition 6 .**

For a right ideal *A* of *S*, a soft set (*F*, *S*) over *U* is called a* union-soft r-ideal* over *U* related to *A* if it satisfies (16) and

(17) $$\begin{array}{c} \left(\forall x\in S\right)\;\left(\forall a\in A\right)\;\left(F\left(ax\right)\subseteq F\left(a\right)\right). \end{array}$$

A union-soft *l*-ideal (resp., union-soft *r*-ideal) over *U* related to *A* = *S* is called a union-soft *l*-ideal (resp., union-soft *r*-ideal) over *U*.

If a soft set (*F*, *S*) over *U* is both a union-soft *l*-ideal and a union-soft *r*-ideal over *U*, we say that (*F*, *S*) is a union-soft ideal over *U*.

**Example 7 .**

Let *S* = {*a*, *b*, *c*, *d*, *e*, *f*} be an ordered semigroup with the following Cayley table and order (see [10]):

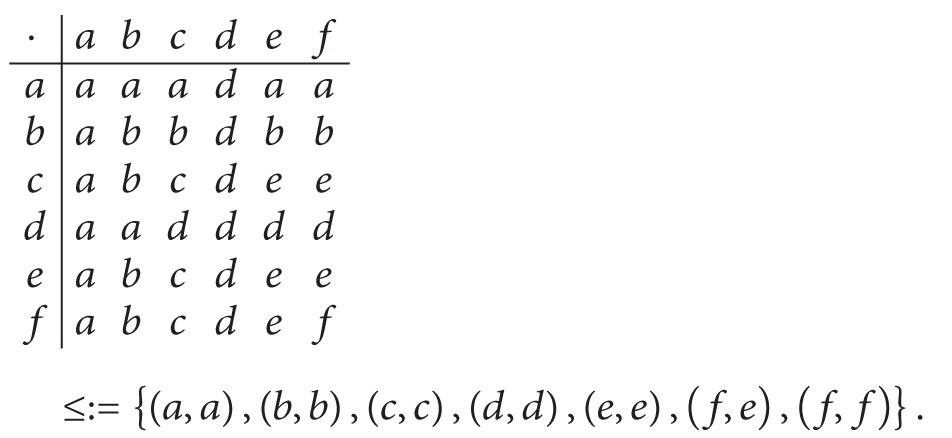
(18)

(1) Let (*F*, *S*) be a soft set over *U* = {1,2, 3,4, 5,6, 7,8, 9,10} in which *F* is given as follows:
  (19) F:S⟶P(U),x⟼{{1,3,5},if  x∈{a,d},{1,2,3,5,6},if  x=b,{1,2,3,5,6,8},if  x∈{c,e,f}.
  Routine calculations show that (*F*, *S*) is a union-soft ideal over *U*.
(2) Let (*G*, *S*) be a soft set over *U* = {1,2, 3,4, 5,6, 7,8, 9,10} in which *G* is given as follows:
  (20) G:S⟶P(U),x⟼{{1,3},if  x=a,{2,8},if  x=d,{1,2,3,8},if  x=b,{1,2,3,8,9},if  x=c,{1,2,3,5,8},if  x∈{e,f}.

Then (*G*, *S*) is a union-soft *l*-ideal over *U*. But it is not a union-soft *r*-ideal over *U* since *G*(*ce*) = *G*(*e*) = {1,2, 3,5, 8} ⊈ {1,2, 3,8, 9} = *G*(*c*).

**Example 8 .**

Let *S* = {*a*, *b*, *c*, *d*, *e*, *f*} be an ordered semigroup with Cayley table and Hasse diagramas shown in Figure 1. In Figure 1, (*F*, *S*) is a soft set over *U* = {1,2, 3,4, 5,6, 7,8, 9,10} in which *F* is given as follows:

(21) F:S⟶P(U),x⟼{{3,5},if  x=a,{1,3,5,6,9},if  x∈{b,d},{1,3,5,6},if  x∈{e,f},{2,4,6,8},if  x=c.

Routine calculations show that (*F*, *S*) is a union-soft *r*-ideal over *U*. But it is not a union-soft *l*-ideal over *U* since *F*(*ce*) = *F*(*c*) = {2,4, 6,8}⊈{1,3, 5,6} = *F*(*e*).

For a nonempty subset *I* of *S*, the* characteristic soft set* is defined to be the soft set (*χ*_*I*_, *S*) over *U* in which *χ*_*I*_ is given as follows:

(22) χI:S⟶P(U),x⟼{U,if  x∈I,∅,otherwise.

The soft set (*U*_*S*_, *S*), where *U*_*S*_(*x*) = *U* for all *x* ∈ *S*, is called the* identity soft set* over *U*. For the characteristic soft set (*χ*_*I*_, *S*) over *U*, the soft set (*χ*_*I*_^*c*^, *S*) over *U* is given as follows:

(23) χIc:S⟶P(U),x⟼{∅,if  x∈I,U,otherwise.

**Theorem 9 .**

For any nonempty subset *I* of *S*, the following are equivalent.

1. *I* is a left (resp., right) ideal of *S*.
2. The soft set (*χ*_*I*_^*c*^, *S*) over *U* is a union-soft *l*-ideal (resp., union-soft *r*-ideal) over *U*.

**Proof**

Assume that *I* is a left ideal of *S*. Let *x*, *y* ∈ *S* be such that *x* ≤ *y*. If *y* ∉ *I*, then *χ*_*I*_^*c*^(*y*) = *U* and so *χ*_*I*_^*c*^(*x*)⊆*U* = *χ*_*I*_^*c*^(*y*). If *y* ∈ *I*, then *χ*_*I*_^*c*^(*y*) = *∅*. Since *x* ≤ *y* and *I* is a left ideal of *S*, we have *x* ∈ *I* and thus *χ*_*I*_^*c*^(*x*) = *∅* = *χ*_*I*_^*c*^(*y*). For any *x*, *y* ∈ *S*, if *y* ∉ *I* then *χ*_*I*_^*c*^(*xy*)⊆*U* = *χ*_*I*_^*c*^(*y*). If *y* ∈ *I*, then *xy* ∈ *I* since *I* is a left ideal of *S*. Hence *χ*_*I*_^*c*^(*xy*) = *∅* = *χ*_*I*_^*c*^(*y*). Therefore (*χ*_*I*_^*c*^, *S*) is a union-soft *l*-ideal over *U*. Similarly, (*χ*_*I*_^*c*^, *S*) is a union-soft *r*-ideal over *U* when *I* is a right ideal of *S*.

Conversely suppose that (*χ*_*I*_^*c*^, *S*) is a union-soft *l*-ideal over *U*. Let *x* ∈ *S* and *y* ∈ *I*. Then *χ*_*I*_^*c*^(*y*) = *∅*, and so *χ*_*I*_^*c*^(*xy*)⊆*χ*_*I*_^*c*^(*y*) = *∅*; that is, *χ*_*I*_^*c*^(*xy*) = *∅*. Thus *xy* ∈ *I*. Let *x* ∈ *S* and *y* ∈ *I* be such that *x* ≤ *y*. Then *χ*_*I*_^*c*^(*x*)⊆*χ*_*I*_^*c*^(*y*) = *∅*, and thus *x* ∈ *I*. Therefore *I* is a left ideal of *S*. Similarly, we can show that if (*χ*_*I*_^*c*^, *S*) is a union-soft *r*-ideal over *U*, then *I* is a right ideal of *S*.

**Corollary 10 .**

For any nonempty subset *I* of *S*, the following are equivalent.

1. *I* is an ideal of *S*.
2. The soft set (*χ*_*I*_^*c*^, *S*) over *U* is a union-soft ideal over *U*.

**Theorem 11 .**

If a soft set (*F*, *S*) over *U* is a union-soft *l*-ideal (resp., union-soft *r*-ideal) over *U*, then the nonempty *δ*-exclusive set of (*F*, *S*) is a left (resp., right) ideal of *S* for all *δ*⊆*U*.

**Proof**

Assume that (*F*, *S*) is a union-soft *l*-ideal over *U*. Let *δ*⊆*U* be such that *e*_*S*_(*F*; *δ*) ≠ *U*. Let *x* ∈ *S* and *y* ∈ *e*_*S*_(*F*; *δ*). Then *F*(*y*)⊆*δ*, and thus *F*(*xy*)⊆*F*(*y*)⊆*δ* by (15). Hence *xy* ∈ *e*_*S*_(*F*; *δ*). Let *x* ∈ *e*_*S*_(*F*; *δ*) and *y* ∈ *S* be such that *y* ≤ *x*. Using (16), we have *F*(*y*)⊆*F*(*x*)⊆*δ*. Thus *y* ∈ *e*_*S*_(*F*; *δ*). Therefore *e*_*S*_(*F*; *δ*) is a left ideal of *S*. The right case can be seen in a similar way.

**Corollary 12 .**

If a soft set (*F*, *S*) over *U* is a union-soft *l*-ideal (resp., union-soft *r*-ideal) over *U*, then the set

(24) $$\begin{array}{c} I_{a}:=\left\{b\in S\;|\;F\left(b\right)\subseteq F\left(a\right)\right\} \end{array}$$

is a left (resp., right) ideal of *S* for every *a* ∈ *S*.

**Theorem 13 .**

Let (*F*, *S*) be a soft set over *U* in which (*Im*⁡(*F*), ⊆) is a chain. If the nonempty *δ*-exclusive set of (*F*, *S*) is a left (resp., right) ideal of *S* for all *δ*⊆*U*, then (*F*, *S*) is a union-soft *l*-ideal (resp., union-soft *r*-ideal) over *U*.

**Proof**

Let *x*, *y* ∈ *S* be such that *x* ≤ *y*. If *F*(*x*)⊋*F*(*y*), then *y* ∈ *e*_*S*_(*F*; *τ*) and *x* ∉ *e*_*S*_(*F*; *τ*) by taking *τ* = *F*(*y*). This is a contradiction, and so *F*(*x*)⊆*F*(*y*). Assume that *F*(*xy*)⊋*F*(*y*) for some *x*, *y* ∈ *S*. Then *y* ∈ *e*_*S*_(*F*; *τ*) and *xy* ∉ *e*_*S*_(*F*; *τ*) by taking *τ* = *F*(*y*). This is a contradiction, and thus *F*(*xy*)⊆*F*(*y*) for all *x*, *y* ∈ *S*. Therefore (*F*, *S*) is a union-soft *l*-ideal over *U*. The right case can be seen in a similar way.

*Question.* In Theorem 13, can we delete the condition “(*Im*⁡(*F*), ⊆) is a chain”?

For any soft sets (*F*, *S*) and (*G*, *S*) over *U*, the union-soft product, denoted by $\left(F,S)\;\tilde{⊙}\;(G,S\right)$, of (*F*, *S*) and (*G*, *S*) is defined to be the soft set $\left(F\;\tilde{⊙}\;G,S\right)$ over *U* in which $F\;\tilde{⊙}\;G$ is a mapping from *S* to *P*(*U*) given by

(25) $$\begin{array}{c} \left(F\;\tilde{⊙}\;G\right)\left(x\right)=\{\begin{array}{cc} \underset{(y,z)\in A_{x}}{⋂}\left\{F\left(y\right)\cup G\left(z\right)\right\}, & \text{if}\;A_{x}\neq \emptyset ,\\ U, & \text{if}\;A_{x}=\emptyset , \end{array} \end{array}$$

where *A*_*x*_ = {(*y*, *z*) ∈ *S* × *S*∣*x* ≤ *yz*}.

**Proposition 14 .**

Let (*χ*_*I*_^*c*^, *S*) and (*χ*_*J*_^*c*^, *S*) be soft sets over *U* where *I* and *J* are nonempty subsets of *S*. Then the following properties hold:

1. $\left(\chi_{I}^{c},S\right)\;\tilde{\cup }\;(\chi_{J}^{c},S)=(\chi_{I\cup J}^{c},S)$.
2. $\left(\chi_{I}^{c},S\right)\;\tilde{⊙}\;(\chi_{J}^{c},S)=(\chi_{\left(IJ\right]}^{c},S)$.

**Proof**

(1) Let *x* ∈ *S*. If *x* ∈ *I* ∪ *J*, then *x* ∈ *I* or *x* ∈ *J*. Thus we have

(26) $$\begin{array}{c} \left(\chi_{I}^{c}\;\tilde{\cup }\;\chi_{J}^{c}\right)\left(x\right)=\chi_{I}^{c}\left(x\right)\cup \chi_{J}^{c}\left(x\right)=\emptyset =\chi_{I\cup J}^{c}\left(x\right). \end{array}$$

If *x* ∉ *I* ∪ *J*, then *x* ∉ *I* and *x* ∉ *J*. Hence we have

(27) $$\begin{array}{c} \left(\chi_{I}^{c}\;\tilde{\cup }\;\chi_{J}^{c}\right)\left(x\right)=\chi_{I}^{c}\left(x\right)\cup \chi_{J}^{c}\left(x\right)=U=\chi_{I\cup J}^{c}\left(x\right). \end{array}$$

Therefore $\left(\chi_{I}^{c},S\right)\;\tilde{\cup }\;\left(\chi_{J}^{c},S\right)=\left(\chi_{I\cup J}^{c},S\right)$.

(2) For any *x* ∈ *S*, suppose *x* ∈ (*IJ*]. Then *x* ≤ *ab* for some *a* ∈ *I* and *b* ∈ *J*, and so (*a*, *b*) ∈ *A*_*x*_. Thus we have

(28) (χIc ⊙~ χJc)(x)=⋂(y,z)∈Ax{χIc(y)∪χJc(z)}⊆χIc(a)∪χJc(b)=∅,

and so $\left(\chi_{I}^{c}\;\tilde{⊙}\;\chi_{J}^{c}\right)(x)=\emptyset$. Since *x* ∈ (*IJ*], we get *χ*_(*IJ*]_^*c*^(*x*) = *∅*. Suppose *x* ∉ (*IJ*]. Then *χ*_(*IJ*]_^*c*^(*x*) = *U*. If *A*_*x*_ = *∅*, then $\left(\chi_{I}^{c}\;\tilde{⊙}\;\chi_{J}^{c}\right)(x)=U$ and $\left(\chi_{I}^{c}\;\tilde{⊙}\;\chi_{J}^{c}\right)(x)=\chi_{\left(IJ\right]}^{c}(x)$. Assume that *A*_*x*_ ≠ *∅*. Then

(29) $$\begin{array}{c} \left(\chi_{I}^{c}\;\tilde{⊙}\;\chi_{J}^{c}\right)\left(x\right)=\underset{(y,z)\in A_{x}}{⋂}\left\{\chi_{I}^{c}\left(y\right)\cup \chi_{J}^{c}\left(z\right)\right\}. \end{array}$$

We now prove that *χ*_*I*_^*c*^(*y*) ∪ *χ*_*J*_^*c*^(*z*) = *U* for all (*y*, *z*) ∈ *A*_*x*_. Let (*y*, *z*) ∈ *A*_*x*_. Then *x* ≤ *yz*. If *y* ∈ *I* and *z* ∈ *J*, then *yz* ∈ *IJ* and so *x* ∈ (*IJ*]. This is impossible. Thus we have *y* ∉ *I* or *z* ∉ *J*. If *y* ∉ *I*, then *χ*_*I*_^*c*^(*y*) = *U* and so *χ*_*I*_^*c*^(*y*) ∪ *χ*_*J*_^*c*^(*z*) = *U*. Similarly, if *z* ∉ *J* then *χ*_*I*_^*c*^(*y*) ∪ *χ*_*J*_^*c*^(*z*) = *U*. In any case, we have $\left(\chi_{I}^{c},S\right)\;\tilde{⊙}\;(\chi_{J}^{c},S)=(\chi_{\left(IJ\right]}^{c},S)$.

**Proposition 15 .**

Let (*F*_1_, *S*), (*F*_2_, *S*), (*G*_1_, *S*), and (*G*_2_, *S*) be soft sets over *U*. If $\left(F_{1},S)\;\tilde{\subseteq }\;(G_{1},S\right)$ and $\left(F_{2},S)\;\tilde{\subseteq }\;(G_{2},S\right)$, then

(30) $$\begin{array}{c} \left(F_{1},S\right)\;\tilde{⊙}\;\left(F_{2},S\right)\;\tilde{\subseteq }\;\left(G_{1},S\right)\;\tilde{⊙}\;\left(G_{2},S\right). \end{array}$$

**Proof**

For any *x* ∈ *S*, if *A*_*x*_ = *∅* then clearly $\left(F_{1},S)\;\tilde{⊙}\;(F_{2},S)\;\tilde{\subseteq }\;(G_{1},S)\;\tilde{⊙}\;(G_{2},S\right)$. Assume that *A*_*x*_ ≠ *∅*. Then

(31) (F1 ⊙~ F2)(x)=⋂(y,z)∈Ax{F1(y)∪F2(z)}⊆⋂(y,z)∈Ax{G1(y)∪G2(z)}=(G1 ⊙~ G2)(x),

and so $\left(F_{1},S)\;\tilde{⊙}\;(F_{2},S)\;\tilde{\subseteq }\;(G_{1},S)\;\tilde{⊙}\;(G_{2},S\right)$.

**Proposition 16 .**

Let (*F*, *S*) and (*G*, *S*) be a union-soft *r*-ideal and a union-soft *l*-ideal over *U*, respectively. Then

(32) $$\begin{array}{c} \left(F,S\right)\;\tilde{\cup }\;\left(G,S\right)\;\tilde{\subseteq }\;\left(F,S\right)\;\tilde{⊙}\;\left(G,S\right). \end{array}$$

**Proof**

Let *x* ∈ *S*. If *A*_*x*_ = *∅*, then $\left(F\;\tilde{⊙}\;G\right)(x)=U⊇\left(F\;\tilde{\cup }\;G\right)(x)$. Suppose that *A*_*x*_ ≠ *∅*. Then $\left(F\;\tilde{⊙}\;G\right)(x)={⋂}_{(y,z)\in A_{x}}\left\{F(y)\cup G(z)\right\}$. Let *y*, *z* ∈ *S* be such that (*y*, *z*) ∈ *A*_*x*_. Then *x* ≤ *yz*. Since (*F*, *S*) is a union-soft *r*-ideal over *U*, it follows that *F*(*x*)⊆*F*(*yz*)⊆*F*(*y*). Since (*G*, *S*) is a union-soft *l*-ideal over *U*, we have *G*(*x*)⊆*G*(*yz*)⊆*G*(*z*). Hence *F*(*x*) ∪ *G*(*x*)⊆*F*(*y*) ∪ *G*(*z*) for all (*y*, *z*) ∈ *A*_*x*_, and so

(33) (F ⊙~ G)(x)=⋂(y,z)∈Ax{F(y)∪G(z)}⊇F(x)∪G(x)=(F ∪~ G)(x).

Therefore $\left(F,S)\;\tilde{⊙}\;(G,S)\;\tilde{⊇}\;(F,S)\;\tilde{\cup }\;(G,S\right)$.

**Proposition 17 .**

Let (*F*, *S*) and (*G*, *S*) be soft sets over *U*. If *S* is regular and (*F*, *S*) is a union-soft *r*-ideal over *U*, then

(34) $$\begin{array}{c} \left(F,S\right)\;\tilde{\cup }\;\left(G,S\right)\;\tilde{⊇}\;\left(F,S\right)\;\tilde{⊙}\;\left(G,S\right). \end{array}$$

**Proof**

Let (*F*, *S*) be a union-soft *r*-ideal over *U*. Let *a* ∈ *S*. Then there exists *x* ∈ *S* such that *a* ≤ (*ax*)*a* since *S* is regular. Thus (*ax*, *a*) ∈ *A*_*a*_; that is, *A*_*a*_ ≠ *∅*, and so

(35) $$\begin{array}{c} \left(F\;\tilde{⊙}\;G\right)\left(a\right)=\underset{(y,z)\in A_{a}}{⋂}\left\{F\left(y\right)\cup G\left(z\right)\right\}. \end{array}$$

On the other hand, since (*F*, *S*) is a union-soft *r*-ideal over *U*, we have

(36) $$\begin{array}{c} F\left(ax\right)\subseteq F\left(a\right). \end{array}$$

Hence $\left(F\;\tilde{\cup }\;G\right)(a)=F(a)\cup G(a)⊇F(ax)\cup G(a)$. Since (*ax*, *a*) ∈ *A*_*a*_, it follows that

(37) (F ∪~ G)(a)⊇F(ax)∪G(a)⊇⋂(y,z)∈Aa{F(y)∪G(z)}=(F ⊙~ G)(a).

Therefore $\left(F,S)\;\tilde{\cup }\;(G,S)\;\tilde{⊇}\;(F,S)\;\tilde{⊙}\;(G,S\right)$.

In a similar way we prove the following.

**Proposition 18 .**

Let (*F*, *S*) and (*G*, *S*) be soft sets over *U*. If *S* is regular and (*G*, *S*) is a union-soft *l*-ideal over *U*, then the soft inclusion (34) is valid.

**Corollary 19 .**

Let (*F*, *S*) and (*G*, *S*) be a union-soft *r*-ideal and a union-soft *l*-ideal over *U*, respectively. If *S* is regular, then

(38) $$\begin{array}{c} \left(F,S\right)\;\tilde{⊙}\;\left(G,S\right)=\left(F,S\right)\;\tilde{\cup }\;\left(G,S\right). \end{array}$$

We now provide a characterization of a regular ordered semigroup.

**Theorem 20 .**

An ordered semigroup *S* is regular if and only if the soft inclusion (34) is valid for every union-soft *r*-ideal (*F*, *S*) and every union-soft *l*-ideal (*G*, *S*) over *U*.

**Proof**

Assume that *S* is regular.  Proposition 17 (or Proposition 18) implies that the soft inclusion (34) is valid.

Conversely, assume that the soft inclusion (34) is valid for every union-soft *r*-ideal (*F*, *S*) and every union-soft *l*-ideal (*G*, *S*) over *U*. Let *a* ∈ *S* and *b* ∈ *R*(*a*)∩*L*(*a*). Since *R*(*a*) (resp., *L*(*a*)) is a right (resp., left) ideal of *S*, it follows from Theorem 9 that (*χ*_*R*(*a*)_^*c*^, *S*) (resp., (*χ*_*L*(*a*)_^*c*^, *S*)) is a union-soft *r*-ideal (resp., union-soft *l*-ideal) over *U*. Using (34), we have

(39) $$\begin{array}{c} \left(\chi_{R(a)}^{c},S\right)\;\tilde{\cup }\;\left(\chi_{L(a)}^{c},S\right)\;\tilde{⊇}\;\left(\chi_{R(a)}^{c},S\right)\;\tilde{⊙}\;\left(\chi_{L(a)}^{c},S\right). \end{array}$$

Since *b* ∈ *R*(*a*)∩*L*(*a*), we have *χ*_*R*(*a*)_^*c*^(*b*) = *∅* = *χ*_*L*(*a*)_^*c*^(*b*). Hence

(40) ∅=χR(a)c(b)∪χL(a)c(b)=(χR(a)c ∪~ χL(a)c)(b)⊇(χR(a)c ⊙~ χL(a)c)(b),

and so $(\chi_{R(a)}^{c}\;\tilde{⊙}\;\chi_{L(a)}^{c})(b)=\emptyset$. Therefore *A*_*b*_ ≠ *∅*. Let *y*, *z* ∈ *S* be such that (*y*, *z*) ∈ *A*_*b*_. Suppose that *y* ∉ *R*(*a*) or *z* ∉ *L*(*a*). Then *χ*_*R*(*a*)_^*c*^(*y*) ∪ *χ*_*L*(*a*)_^*c*^(*z*) = *U*, and so

(41) $$\begin{array}{c} \left(\chi_{R(a)}^{c}\;\tilde{⊙}\;\chi_{L(a)}^{c}\right)\left(b\right)=\underset{(y,z)\in A_{b}}{⋂}\left\{\chi_{R(a)}^{c}\left(y\right)\cup \chi_{L(a)}^{c}\left(z\right)\right\}=U. \end{array}$$

This is a contradiction, and thus *y* ∈ *R*(*a*) and *z* ∈ *L*(*a*). Therefore *b* ≤ *yz* ∈ *R*(*a*)*L*(*a*), which implies that *b* ∈ (*R*(*a*)*L*(*a*)]. Hence

(42) $$\begin{array}{c} R\left(a\right)\cap L\left(a\right)\subseteq \left(R\left(a\right)L\left(a\right)\right]. \end{array}$$

Using Lemma 1, we know that *S* is regular.

**Theorem 21 .**

For a union-soft *l*-ideal (*G*, *S*) over *U*, the following assertion is valid

(43) $$\begin{array}{c} \left(\emptyset_{S},S\right)\;\tilde{⊙}\;\left(G,S\right)\;\tilde{⊇}\;\left(G,S\right), \end{array}$$

where *∅*_*S*_ is an empty soft set over *U*; that is, *∅*_*S*_(*x*) = *∅* for all *x* ∈ *S*.

**Proof**

Suppose that (*G*, *S*) is a union-soft *l*-ideal over *U*. Let *x* ∈ *S*. If *A*_*x*_ ≠ *∅*, then

(44) (∅S ⊙~ G)(x)=⋂(y,z)∈Ax{∅S(y)∪G(z)}⊇⋂(y,z)∈Ax{∅∪G(yz)}⊇G(x).

Assume that *A*_*x*_ = *∅*. Then $\left(\emptyset_{S}\;\tilde{⊙}\;G\right)(x)=U⊇G(x)$. This completes the proof.

**Theorem 22 .**

Let (*∅*_*S*_, *S*) be the empty soft set over *U* and (*G*, *S*) be a soft set over *U*. If (*G*, *S*) satisfies the conditions (43) and (16), then (*G*, *S*) is a union-soft *l*-ideal over *U*.

**Proof**

For any *x*, *y* ∈ *S*, we have

(45) G(xy)⊆(∅S ⊙~ G)(xy)=⋂(x,y)∈Axy{∅S(x)∪G(y)}⊆∅S(x)∪G(y)=∅∪G(y)=G(y).

Hence (*G*, *S*) is a union-soft *l*-ideal over *U*.

Similarly, we have the following theorem.

**Theorem 23 .**

For the empty soft set (*∅*_*S*_, *S*) over *U* and a soft set (*G*, *S*) over *U*, the following assertions are equivalent:

(1) (*G*, *S*) is a union-soft *r*-ideal over *U*.
(2) (*G*, *S*) satisfies the conditions (16) and
  (46) $$\begin{array}{c} \left(G,S\right)\;\tilde{⊙}\;\left(\emptyset_{S},S\right)\;\tilde{⊇}\;\left(G,S\right). \end{array}$$

**Corollary 24 .**

For the empty soft set (*∅*_*S*_, *S*) over *U* and a soft set (*G*, *S*) over *U*, the following assertions are equivalent:

(1) (*G*, *S*) is a union-soft ideal over *U*.
(2) (*G*, *S*) satisfies the conditions (16) and
  (47) $$\begin{array}{c} \left(\emptyset_{S},S\right)\;\tilde{⊙}\;\left(G,S\right)\;\tilde{⊇}\;\left(G,S\right),\;\;\left(G,S\right)\;\tilde{⊙}\;\left(\emptyset_{S},S\right)\;\tilde{⊇}\;\left(G,S\right). \end{array}$$

**Lemma 25 .**

If (*F*, *S*) (resp., (*G*, *S*)) is a union-soft *r*-ideal (resp., union-soft *l*-ideal) over *U*, then

1. $\left(F,S)\;\tilde{⊙}\;\left(\emptyset_{S},S\right)\;\tilde{⊇}\;(F,S\right)$ (resp., $\left(\emptyset_{S},S\right)\;\tilde{⊙}\;(G,S)\;\tilde{⊇}\;(G,S)$).
2. $\left(F,S)\;\tilde{⊙}\;(F,S)\;\tilde{⊇}\;(F,S\right)$ (resp., $\left(G,S)\;\tilde{⊙}\;(G,S)\;\tilde{⊇}\;(G,S\right)$).

**Proof**

(1) Assume that (*F*, *S*) is a union-soft *r*-ideal over *U* and let *x* ∈ *S*. If *A*_*x*_ = *∅*, then $\left(F\;\tilde{⊙}\;\emptyset_{S}\right)(x)=U⊇F(x)$. Assume that *A*_*x*_ ≠ *∅* and let *y*, *z* ∈ *S* be such that (*y*, *z*) ∈ *A*_*x*_. Then *x* ≤ *yz*, and so *F*(*x*)⊆*F*(*yz*)⊆*F*(*y*) by (16) and (17). Hence *F*(*y*) ∪ *∅*_*S*_(*z*) = *F*(*y*) ∪ *∅* = *F*(*y*)⊇*F*(*x*) for all (*y*, *z*) ∈ *A*_*x*_. Therefore

(48) $$\begin{array}{c} \left(F\;\tilde{⊙}\;\emptyset_{S}\right)\left(x\right)=\underset{(y,z)\in A_{x}}{⋂}\left\{F\left(y\right)\cup \emptyset_{S}\left(z\right)\right\}⊇F\left(x\right). \end{array}$$

Consequently, $\left(F,S)\;\tilde{⊙}\;\left(\emptyset_{S},S\right)\;\tilde{⊇}\;(F,S\right)$. Similarly, $\left(\emptyset_{S},S\right)\;\tilde{⊙}\;(G,S)\;\tilde{⊇}\;(G,S)$ for each union-soft *l*-ideal (*G*, *S*) over *U*.

(2) Note that $(F,S)\;\tilde{⊇}\;\left(\emptyset_{S},S\right)$ and $\left(F,S)\;\tilde{\subseteq }\;(F,S\right)$. Using Proposition 15 and (1), we have $\left(F,S)\;\tilde{⊙}\;(F,S)\;\tilde{⊇}\;(F,S)\;\tilde{⊙}\;\left(\emptyset_{S},S\right)\;\tilde{⊇}\;(F,S\right)$. In a similar way, we obtain

(49) $$\begin{array}{c} \left(G,S\right)\;\tilde{⊙}\;\left(G,S\right)\;\tilde{⊇}\;\left(G,S\right) \end{array}$$

for each union-soft *l*-ideal (*G*, *S*) over *U*.

**Proposition 26 .**

Let *S* be a regular ordered semigroup. If (*F*, *S*) (resp., (*G*, *S*)) is a union-soft *r*-ideal (resp., union-soft *l*-ideal) over *U*, then

(50) $$\begin{array}{c} \left(F,S\right)\;\tilde{⊇}\;\left(F,S\right)\;\tilde{⊙}\;\left(F,S\right)\;\;\left(resp.\;\;\left(G,S\right)\;\tilde{⊇}\;\left(G,S\right)\;\tilde{⊙}\;\left(G,S\right)\right). \end{array}$$

**Proof**

Let (*F*, *S*) be a union-soft *r*-ideal over *U* and let *a* ∈ *S*. Then there exists *x* ∈ *S* such that *a* ≤ *ax* *a*. Hence *A*_*a*_ ≠ *∅* since (*ax*, *a*) ∈ *A*_*a*_. Thus

(51) (F ⊙~ F)(a)=⋂(y,z)∈Aa{F(y)∪F(z)}⊆F(ax)∪F(a)=F(a)

since *F*(*a*)⊆*F*((*ax*)*a*)⊆*F*(*ax*)⊆*F*(*a*). Therefore $\left(F,S)\;\tilde{⊇}\;(F,S)\;\tilde{⊙}\;(F,S\right)$. Similarly we have $\left(G,S)\;\tilde{⊇}\;(G,S)\;\tilde{⊙}\;(G,S\right)$ for each union-soft *l*-ideal (*G*, *S*) over *U*.

We say that a soft set (*F*, *S*) over *U* is* soft idempotent* if $\left(F,S)\;\tilde{⊙}\;(F,S)=(F,S\right)$.

By Lemma 25(2) and Proposition 26 we have the following result.

**Proposition 27 .**

If *S* is a regular ordered semigroup, then every union-soft *r*-ideal (resp., union-soft *l*-ideal) over *U* is soft idempotent.

**Definition 28 .**

A soft set (*F*, *S*) over *U* is said to be union-soft semiprime if it satisfies

(52) $$\begin{array}{c} \left(\forall x\in S\right)\;\left(F\left(x\right)\subseteq F\left(x^{2}\right)\right). \end{array}$$

**Theorem 29 .**

If *S* is left regular, then every union-soft *l*-ideal is a union-soft semiprime.

**Proof**

Let (*F*, *S*) be a union-soft *l*-ideal over *U* and let *a* ∈ *S*. Then *a* ≤ *xa* ^2^ for some *x* ∈ *S* since *S* is left regular. It follows from (16) and (15) that

(53) $$\begin{array}{c} F\left(a\right)\subseteq F\left(xa^{2}\right)\subseteq F\left(a^{2}\right). \end{array}$$

Hence (*F*, *S*) is union-soft semiprime.

In a similar way, we have the following theorem.

**Theorem 30 .**

If *S* is right regular, then every union-soft *r*-ideal is union-soft semiprime.

**Theorem 31 .**

If *S* is intraregular, then every union-soft ideal is union-soft semiprime.

**Proof**

Let (*F*, *S*) be a union-soft ideal over *U* and let *a* ∈ *S*. Then *a* ≤ *xa* ^2^ *y* for some *x*, *y* ∈ *S* since *S* is intraregular. It follows from (16), (15), and (17) that

(54) $$\begin{array}{c} F\left(a\right)\subseteq F\left(xa^{2}y\right)\subseteq F\left(a^{2}y\right)\subseteq F\left(a^{2}\right). \end{array}$$

Hence (*F*, *S*) is union-soft semiprime.

**Corollary 32 .**

If *S* is intraregular, then every union-soft ideal (*F*, *S*) over *U* satisfies the following equality:

(55) $$\begin{array}{c} \left(\forall x,y\in S\right)\;\left(F\left(xy\right)=F\left(yx\right)\right). \end{array}$$

**Proof**

Using Theorem 31, we have

(56) $$\begin{array}{c} F\left(xy\right)\subseteq F\left({\left(xy\right)}^{2}\right)=F\left(x\left(yx\right)y\right)\subseteq F\left(yx\right),\\ F\left(yx\right)\subseteq F\left({\left(yx\right)}^{2}\right)=F\left(y\left(xy\right)x\right)\subseteq F\left(xy\right) \end{array}$$

for all *x*, *y* ∈ *S*. This completes the proof.

## 4. Concave Soft Sets and Critical Soft Points

For any soft set (*F*, *S*) over *U*, consider a soft set ([[*F*]], *S*) over *U* where

(57) $$\begin{array}{c} \left[\left[F\right]\right]:S⟶P\left(U\right),\;x⟼\underset{x\leq y}{⋂}F\left(y\right). \end{array}$$

Since *x* ≤ *x* for all *x* ∈ *S*, we have

(58) $$\begin{array}{c} \left[\left[F\right]\right]\left(x\right)=\underset{x\leq y}{⋂}F\left(y\right)\subseteq F\left(x\right) \end{array}$$

for all *x* ∈ *S*. Hence $\left(F,S)\;\tilde{⊇}\;([[F]],S\right)$.

A soft set (*F*, *S*) over *U* is said to be concave if $\left([[F]],S)\;\tilde{⊇}\;(F,S\right)$, and hence (*F*, *S*) = ([[*F*]], *S*).

**Theorem 33 .**

For a soft set (*F*, *S*) over *U*, the following are equivalent:

1. (*F*, *S*) is concave.
2. (∀*x*, *y* ∈ *S*)  (*x* ≤ *y*⇒*F*(*x*)⊆*F*(*y*)).

**Proof**

Assume that (*F*, *S*) is concave. Let *x*, *y* ∈ *S* be such that *x* ≤ *y*. Then

(59) $$\begin{array}{c} F\left(x\right)=\left[\left[F\right]\right]\left(x\right)=\underset{x\leq w}{⋂}F\left(w\right)\subseteq F\left(y\right). \end{array}$$

Conversely, if (2) is valid, then [[*F*]](*x*) = ⋂_*x*≤*y*_ *F*(*y*)⊇*F*(*x*) for all *x* ∈ *S*. Hence $\left([[F]],S)\;\tilde{⊇}\;(F,S\right)$; that is, (*F*, *S*) is concave.

**Proposition 34 .**

For any soft sets (*F*, *S*), (*G*, *S*), and (*H*, *S*) over *U*, we have

1. if $\left(F,S)\;\tilde{\subseteq }\;(G,S\right)$, then $\left([[F]],S)\;\tilde{\subseteq }\;([[G]],S\right)$.
2. $\left([[F]],S)\;\tilde{⊙}\;([[G]],S)\;\tilde{⊇}\;([[F\;\tilde{⊙}\;G]],S\right)$.
3. ([[*F*]], *S*) is concave.

**Proof**

(1) If $\left(F,S)\;\tilde{\subseteq }\;(G,S\right)$, then *F*(*x*)⊆*G*(*x*) for all *x* ∈ *S*. Thus

(60) $$\begin{array}{c} \left[\left[F\right]\right]\left(x\right)=\underset{x\leq y}{⋂}F\left(y\right)\subseteq \underset{x\leq y}{⋂}G\left(y\right)=\left[\left[G\right]\right]\left(x\right) \end{array}$$

for all *x* ∈ *S*. Therefore $\left([[F]],S)\;\tilde{\subseteq }\;([[G]],S\right)$.

(2) Let *x* ∈ *S*. If *A*_*x*_ = *∅*, then $\left(\left[[F]]\;\tilde{⊙}\;[[G]\right]\right)(x)=U⊇[[F\;\tilde{⊙}\;G]](x)$. If *A*_*x*_ ≠ *∅*, then *x* ≤ *yz* for some *y*, *z* ∈ *S*. Thus

(61) ([[F]] ⊙~ [[G]])(x)=⋂(y,z)∈Ax{[[F]](y)∪[[G]](z)}=⋂(y,z)∈Ax{(⋂y≤sF(s))∪(⋂z≤tG(t))}=⋂(y,z)∈Ax{⋂y≤s,z≤t{F(s)∪G(t)}}⊇⋂(y,z)∈Ax{⋂yz≤st{F(s)∪G(t)}}=⋂x≤yz(F ⊙~ G)(yz)=[[F ⊙~ G]](x).

Therefore $\left([[F]],S)\;\tilde{⊙}\;([[G]],S)\;\tilde{⊇}\;([[F\;\tilde{⊙}\;G]],S\right)$.

(3) Let *x*, *y* ∈ *S* be such that *x* ≤ *y*. Then

(62) $$\begin{array}{c} \left[\left[F\right]\right]\left(y\right)=\underset{y\leq z}{⋂}F\left(z\right)⊇\underset{x\leq z}{⋂}F\left(z\right)=\left[\left[F\right]\right]\left(x\right). \end{array}$$

It follows from Theorem 33 that ([[*F*]], *S*) is concave.

Let (*F*, *S*) be a soft set over *U*. For any *a* ∈ *S* and any proper subset *λ* of *U*, a* critical soft point*, denoted by ((*a*]_*λ*_, *S*), over *U* is defined to be a soft set over *U* where

(63) $$\begin{array}{c} {\left(a\right]}_{\lambda }:S⟶P\left(U\right),\;x⟼\{\begin{array}{cc} \lambda , & \text{if}\;x\in \left(a\right],\\ U, & \text{otherwise}. \end{array} \end{array}$$

**Proposition 35 .**

For any proper subsets *λ* and *δ* of *U*, if ((*a*]_*λ*_, *S*) and ((*b*]_*δ*_, *S*) are critical soft points over *U*, then $\left({\left(a\right]}_{\lambda }\;\tilde{⊙}\;{\left(b\right]}_{\delta },S\right)=\left({\left(ab\right]}_{\lambda \cup \delta },S\right)$.

**Proof**

Let *x* ∈ *S*. If *x* ∈ (*ab*], then *A*_*x*_ ≠ *∅* and so

(64) ((a]λ ⊙~ (b]δ)(x) =⋂(y,z)∈Ax{(a]λ(y)∪(b]δ(z)} ⊆(a]λ(a)∪(b]δ(b)=λ∪δ.

Note that (*a*]_*λ*_(*y*)∪(*b*]_*δ*_(*z*)⊇*λ* ∪ *δ* for all *y*, *z* ∈ *S*. Hence $\left({\left(a\right]}_{\lambda }\;\tilde{⊙}\;{\left(b\right]}_{\delta }\right)(x)⊇\lambda \cup \delta$. It follows that

(65) $$\begin{array}{c} \left({\left(a\right]}_{\lambda }\;\tilde{⊙}\;{\left(b\right]}_{\delta }\right)\left(x\right)=\lambda \cup \delta ={\left(ab\right]}_{\lambda \cup \delta }\left(x\right). \end{array}$$

For *x* ∉ (*ab*], assume that $\left({\left(a\right]}_{\lambda }\;\tilde{⊙}\;{\left(b\right]}_{\delta }\right)(x)\neq U$. Then

(66) ((a]λ ⊙~ (b]δ)(x)=⋂(y,z)∈Ax{(a]λ(y)∪(b]δ(z)}≠U,

and so (*a*]_*λ*_(*y*_0_)∪(*b*]_*δ*_(*z*_0_) ≠ *U* for some *y*_0_, *z*_0_ ∈ *S* with *x* ≤ *y*_0_ *z*_0_. Thus (*a*]_*λ*_(*y*_0_) = *λ* and (*b*]_*δ*_(*z*_0_) = *δ*; that is, *y*_0_ ∈ (*a*] and *z*_0_ ∈ (*b*]. It follows that *y*_0_ *z*_0_⊆(*a*](*b*]⊆(*ab*] and that *x* ∈ (*ab*]. This is a contradiction. Therefore $\left({\left(a\right]}_{\lambda }\;\tilde{⊙}\;{\left(b\right]}_{\delta }\right)(x)=U={\left(ab\right]}_{\lambda \cup \delta }(x)$. Consequently, we know that

(67) $$\begin{array}{c} \left({\left(a\right]}_{\lambda }\;\tilde{⊙}\;{\left(b\right]}_{\delta }\right)\left(x\right)={\left(ab\right]}_{\lambda \cup \delta }\left(x\right) \end{array}$$

for all *x* ∈ *S*; that is, $\left({\left(a\right]}_{\lambda }\;\tilde{⊙}\;{\left(b\right]}_{\delta },S\right)=\left({\left(ab\right]}_{\lambda \cup \delta },S\right)$.

**Corollary 36 .**

For any proper subsets *λ* and *δ* of *U*, if ((*a*]_*λ*_, *S*) and ((*b*]_*δ*_, *S*) are critical soft points over *U*, then

(68) $$\begin{array}{c} \left({\left(a\right]}_{\lambda }\;\tilde{⊙}\;{\left(b\right]}_{\delta },S\right)=\left({\left(b\right]}_{\delta }\;\tilde{⊙}\;{\left(a\right]}_{\lambda },S\right)⟺ab=ba. \end{array}$$

**Proof**

It is straightforward.

**Proposition 37 .**

If (*F*, *S*) is a concave soft set over *U*, then

(69) $$\begin{array}{c} \left(F,S\right)=\left(\overset{\sim }{\underset{\left(F,S)\tilde{\subseteq }({\left(a\right]}_{\lambda },S\right)}{⋂}}{\left(a\right]}_{\lambda },S\right) \end{array}$$

for any *a* ∈ *S* and a proper subset *λ* of *U*.

**Proof**

Let ((*a*]_*λ*_, *S*) be a critical soft point over *U* such that $\left(F,S)\;\tilde{\subseteq }\;({\left(a\right]}_{\lambda },S\right)$. Then (*a*]_*λ*_(*x*)⊇*F*(*x*) for all *x* ∈ *S*, and so

(70) (⋂(F,S)⊆~((a]λ,S)~(a]λ)(x)=⋂(F,S)⊆~((a]λ,S)(a]λ(x)⊇⋂(F,S)⊆~((a]λ,S)F(x)=F(x)

for all *x* ∈ *S*. Hence

(71) $$\begin{array}{c} \left(\overset{\sim }{\underset{\left(F,S)\tilde{\subseteq }({\left(a\right]}_{\lambda },S\right)}{⋂}}{\left(a\right]}_{\lambda },S\right)\;\tilde{⊇}\;\left(F,S\right). \end{array}$$

On the other hand, let *F*(*x*) = *λ* for *x* ∈ *S*. Then $\left(F,S)\;\tilde{\subseteq }\;(x_{\lambda },S\right)$. In fact, if *y* ∉ (*x*] then *x*_*λ*_(*y*) = *U*⊇*F*(*y*). If *y* ∈ (*x*], then *y* ≤ *x* and *x*_*λ*_(*y*) = *λ*. Since (*F*, *S*) is concave, it follows from Theorem 33 that *F*(*y*)⊆*F*(*x*) = *λ* = *x*_*λ*_(*y*). Therefore $\left(x_{\lambda },S)\;\tilde{⊇}\;(F,S\right)$, and so

(72) $$\begin{array}{c} F\left(x\right)=\lambda =x_{\lambda }\left(x\right)⊇\left(\underset{\left(F,S)\tilde{\subseteq }({\left(a\right]}_{\lambda },S\right)}{⋂}{\left(a\right]}_{\lambda }\right)\left(x\right). \end{array}$$

Hence

(73) $$\begin{array}{c} \left(F,S\right)\;\tilde{⊇}\;\left(\overset{\sim }{\underset{\left(F,S)\tilde{\subseteq }({\left(a\right]}_{\lambda },S\right)}{⋂}}{\left(a\right]}_{\lambda },S\right). \end{array}$$

**Theorem 38 .**

For any soft sets (*F*, *S*), (*G*, *S*), and (*H*, *S*) over *U*, the following items are valid:

1. if (*F*, *S*) is a union-soft ideal over *U*, then it is concave; that is, (*F*, *S*) = ([[*F*]], *S*);
2. if (*F*, *S*) and (*G*, *S*) are union-soft *l*-ideals (resp., union-soft *r*-ideals) over *U*, then so are $\left(F\;\tilde{⊙}\;G,S\right)$ and $\left(F\;\tilde{\cap }\;G,S\right)$.

**Proof**

(1) Since every union-soft ideal (*F*, *S*) over *U* satisfies the condition (16), it follows from Theorem 33.

(2) Assume that (*F*, *S*) and (*G*, *S*) are union-soft *l*-ideals over *U*. For any *x*, *y* ∈ *S* with *x* ≤ *y*, we have

(74) (F ⊙~ G)(x)=⋂(a,b)∈Ax{F(a)∪G(b)}⊆⋂(a,b)∈Ay{F(a)∪G(b)}=(F ⊙~ G)(y).

Theorem 21 implies that

(75) (∅S,S) ⊙~ ((F,S) ⊙~ (G,S))  =((∅S,S) ⊙~ (F,S)) ⊙~ (G,S)  ⊇~ (F,S) ⊙~ (G,S).

It follows from Theorem 22 that $\left(F\;\tilde{⊙}\;G,S\right)$ is a union-soft *l*-ideal over *U*.

It is easy to verify that $\left(\emptyset_{S},S)\;\tilde{⊙}\;((F,S)\;\tilde{\cap }\;(G,S))\;\tilde{⊇}\;(F,S)\;\tilde{\cap }\;(G,S\right)$. Let *x*, *y* ∈ *S* be such that *x* ≤ *y*. Then *F*(*x*)⊆*F*(*y*) and *G*(*x*)⊆*G*(*y*). Hence

(76) (F ∩~ G)(x)=F(x)∩G(x)⊆F(y)∩G(y)=(F ∩~ G)(y).

Therefore $\left(F\;\tilde{\cap }\;G,S\right)$ is a union-soft *l*-ideal over *U* by Theorem 22. Similarly, one can prove that $\left(F\;\tilde{⊙}\;G,S\right)$ and $\left(F\;\tilde{\cap }\;G,S\right)$ are union-soft *r*-ideals over *U* when (*F*, *S*) and (*G*, *S*) are union-soft *r*-ideals over *U*.

**Theorem 39 .**

For any critical soft point ((*a*]_*λ*_, *S*) over *U*, let (*F*_*l*_((*a*]_*λ*_), *S*) be a soft set over *U* in which *F*_*l*_((*a*]_*λ*_) is given as follows:

(77) Fl((a]λ):S⟶P(U),x⟼{λ,if  x∈L(a),U,otherwise.

Then (*F*_*l*_((*a*]_*λ*_), *S*) is the greatest union-soft *l*-ideal over *U* which is contained in the critical soft point ((*a*]_*λ*_, *S*).

**Proof**

Let *x*, *y* ∈ *S*. If *F*_*l*_((*a*]_*λ*_)(*y*) = *U*, then it is clear that *F*_*l*_((*a*]_*λ*_)(*xy*)⊆*F*_*l*_((*a*]_*λ*_)(*y*). If *F*_*l*_((*a*]_*λ*_)(*y*) ≠ *U*, then *F*_*l*_((*a*]_*λ*_)(*y*) = *λ* and *y* ∈ (*S* ^1^ *a*]. Thus *y* ≤ *ba* for some *b* ∈ *S* ^1^, and so *xy* ≤ (*xb*)*a*. Hence *xy* ∈ *L*(*a*), and thus *F*_*l*_((*a*]_*λ*_)(*xy*) = *λ*⊆*F*_*l*_((*a*]_*λ*_)(*y*). Assume that *x* ≤ *y*. If *y* ∉ *L*(*a*) then *F*_*l*_((*a*]_*λ*_)(*y*) = *U*⊇*F*_*l*_((*a*]_*λ*_)(*x*). If *y* ∈ *L*(*a*) then *x* ∈ *L*(*a*) since *x* ≤ *y*. Thus *F*_*l*_((*a*]_*λ*_)(*x*) = *λ*⊆*F*_*l*_((*a*]_*λ*_)(*y*). Consequently, (*F*_*l*_((*a*]_*λ*_), *S*) is a union-soft *l*-ideal over *U*. For each *x* ∈ *S*, if *x* ∈ (*a*] then *x* ∈ *L*(*a*) and so (*a*]_*λ*_(*x*) = *λ* = *F*_*l*_((*a*]_*λ*_)(*x*). If *x* ∉ (*a*] then (*a*]_*λ*_(*x*) = *U*⊇*F*_*l*_((*a*]_*λ*_)(*x*). Therefore $\left({\left(a\right]}_{\lambda },S)\;\tilde{⊇}\;(F_{l}({\left(a\right]}_{\lambda }),S\right)$. Let (*G*, *S*) be a union-soft *l*-ideal over *U* such that $\left({\left(a\right]}_{\lambda },S)\;\tilde{⊇}\;(G,S\right)$. If *x* ∈ *L*(*a*), then there exists *b* ∈ *S* ^1^ such that *x* ≤ *ba*. Hence

(78) $$\begin{array}{c} F_{l}\left({\left(a\right]}_{\lambda }\right)\left(x\right)=\lambda ={\left(a\right]}_{\lambda }\left(a\right)⊇G\left(a\right)⊇G\left(ba\right)⊇G\left(x\right). \end{array}$$

If *x* ∉ *L*(*a*), then *G*(*x*)⊆*U* = *F*_*l*_((*a*]_*λ*_)(*x*). Therefore $\left(G,S)\;\tilde{\subseteq }\;(F_{l}({\left(a\right]}_{\lambda }),S\right)$. This completes the proof.

Similarly, we have the following theorem.

**Theorem 40 .**

For any critical soft point ((*a*]_*λ*_, *S*) over *U*, let (*F*_*r*_((*a*]_*λ*_), *S*) be a soft set over *U* in which *F*_*r*_((*a*]_*λ*_) is given as follows:

(79) Fr((a]λ):S⟶P(U),x⟼{λ,if  x∈R(a),U,otherwise.

Then (*F*_*r*_((*a*]_*λ*_), *S*) is the greatest union-soft *r*-ideal over *U* which is contained in the critical soft point ((*a*]_*λ*_, *S*).

**Theorem 41 .**

Let ((*a*]_*λ*_, *S*) be a critical soft points over *U*. Then $\left(\emptyset_{S},S)\;\tilde{⊙}\;({\left(a\right]}_{\lambda },S)\;\tilde{⊙}\;(\emptyset_{S},S\right)$ is a union-soft ideal over *U*, and

(80) (∅S ⊙~ (a]λ ⊙~ ∅S)(x)={λ,if  x∈(SaS],U,if  x∉(SaS],

for all *x* ∈ *S*.

**Proof**

Let *x* ∈ *S*. If *x* ∈ (*S* *aS*], then there exist *y*, *z* ∈ *S* such that *x* ≤ *yaz*. Hence

(81) (∅S ⊙~ (a]λ ⊙~ ∅S)(x)  =⋂x≤x1x2x3{∅S(x1)∪(a]λ(x2)∪∅S(x3)}  ⊆∅S(y)∪(a]λ(a)∪∅S(z)  =∅∪λ∪∅=λ.

On the other hand, *∅*_*S*_(*x*_1_)∪(*a*]_*λ*_(*x*_2_) ∪ *∅*_*S*_(*x*_3_) = (*a*]_*λ*_(*x*_2_)⊇*λ* for all *x*_1_, *x*_2_, *x*_3_ ∈ *S*, and so

(82) (∅S ⊙~ (a]λ ⊙~ ∅S)(x)  =⋂x≤x1x2x3{∅S(x1)∪(a]λ(x2)∪∅S(x3)}⊇λ

for all *x* ∈ *S*. It follows that $(\emptyset_{S}\;\tilde{⊙}\;{\left(a\right]}_{\lambda }\;\tilde{⊙}\;\emptyset_{S})(x)=\lambda$ for all *x* ∈ (*S* *aS*]. Assume that *x* ∉ (*S* *aS*]. If there exist *x*_1_, *x*_2_, *x*_3_ ∈ *S* such that *x* ≤ *x*_1_ *x*_2_ *x*_3_, then

(83) (∅S ⊙~ (a]λ ⊙~ ∅S)(x)  =⋂x≤x1x2x3{∅S(x1)∪(a]λ(x2)∪∅S(x3)}  =⋂x≤x1x2x3(a]λ(x2).

If $(\emptyset_{S}\;\tilde{⊙}\;{\left(a\right]}_{\lambda }\;\tilde{⊙}\;\emptyset_{S})(x)\neq U$, then there exist *a*, *b*, *c* ∈ *S* such that *x* ≤ *ab* *c* and *b* ∈ (*a*]. Thus *x* ∈ (*S* *aS*] which leads a contradiction. Therefore $(\emptyset_{S}\;\tilde{⊙}\;{\left(a\right]}_{\lambda }\;\tilde{⊙}\;\emptyset_{S})(x)=U$. If there does not exist *x*_1_, *x*_2_, *x*_3_ ∈ *S* such that *x* ≤ *x*_1_ *x*_2_ *x*_3_, then $(\emptyset_{S}\;\tilde{⊙}\;{\left(a\right]}_{\lambda }\;\tilde{⊙}\;\emptyset_{S})(x)=U$. Now, it is easy to verify that

(84) (∅S,S) ⊙~ (∅S ⊙~ (a]λ ⊙~ ∅S,S) ⊇~ (∅S ⊙~ (a]λ ⊙~ ∅S,S),(∅S ⊙~ (a]λ ⊙~ ∅S,S) ⊙~ (∅S,S) ⊇~ (∅S ⊙~ (a]λ ⊙~ ∅S,S).

Let *x*, *y* ∈ *S* be such that *x* ≤ *y*. Obviously, $(\emptyset_{S}\;\tilde{⊙}\;\;{\left(a\right]}_{\lambda }\;\;\tilde{⊙}\;\;\emptyset_{S})(x)⊇\lambda$ for all *x* ∈ *S*. If *x* ∈ (*S* *aS*], then $\left(\emptyset_{S}\;\tilde{⊙}\;{\left(a\right]}_{\lambda }\;\tilde{⊙}\;\emptyset_{S})(x)=\lambda \subseteq (\emptyset_{S}\;\tilde{⊙}\;{\left(a\right]}_{\lambda }\;\tilde{⊙}\;\emptyset_{S})(y\right)$. If *x* ∉ (*S* *aS*], then *y* ∉ (*S* *aS*] and so $\left(\emptyset_{S}\;\tilde{⊙}\;{\left(a\right]}_{\lambda }\;\tilde{⊙}\;\emptyset_{S})(x)=U=(\emptyset_{S}\;\tilde{⊙}\;{\left(a\right]}_{\lambda }\;\tilde{⊙}\;\emptyset_{S})(y\right)$. Therefore $\left(\emptyset_{S},S)\;\tilde{⊙}\;({\left(a\right]}_{\lambda },S)\;\tilde{⊙}\;(\emptyset_{S},S\right)$ is a union-soft ideal over *U* by Theorems 22 and 23.

Similarly, we have the following theorems.

**Theorem 42 .**

Let ((*a*]_*λ*_, *S*) be a critical soft points over *U*. Then $\left(\emptyset_{S},S)\;\tilde{⊙}\;{\left((a\right]}_{\lambda },S\right)$ is a union-soft *l*-ideal over *U*, and

(85) $$\begin{array}{c} \left(\emptyset_{S}\;\tilde{⊙}\;{\left(a\right]}_{\lambda }\right)\left(x\right)=\{\begin{array}{cc} \lambda , & if\;x\in \left(Sa\right],\\ U, & if\;x\notin \left(Sa\right], \end{array} \end{array}$$

for all *x* ∈ *S*.

**Theorem 43 .**

Let ((*a*]_*λ*_, *S*) be critical soft points over *U*. Then $\left({\left(a\right]}_{\lambda },S)\;\tilde{⊙}\;(\emptyset_{S},S\right)$ is a union-soft *r*-ideal over *U*, and

(86) $$\begin{array}{c} \left({\left(a\right]}_{\lambda }\;\tilde{⊙}\;\emptyset_{S}\right)\left(x\right)=\{\begin{array}{cc} \lambda , & if\;x\in \left(aS\right],\\ U, & if\;x\notin \left(aS\right], \end{array} \end{array}$$

for all *x* ∈ *S*.

**Proposition 44 .**

For any critical soft points ((*a*]_*λ*_, *S*) and ((*b*]_*δ*_, *S*) over *U*, we have

(87) (∅S,S) ⊙~ ((a]λ,S) ⊙~ (∅S,S) ⊆~ ((b]δ,S)⟺b∈(SaS],δ⊆λ.

**Proof**

If $\left(\emptyset_{S},S)\;\tilde{⊙}\;({\left(a\right]}_{\lambda },S)\;\tilde{⊙}\;(\emptyset_{S},S)\;\tilde{\subseteq }\;({\left(b\right]}_{\delta },S\right)$, then $\left(\emptyset_{S}\;\tilde{⊙}\;{\left(a\right]}_{\lambda }\;\tilde{⊙}\;\emptyset_{S}\right)(b)\subseteq {\left(b\right]}_{\delta }(b)=\delta \neq U$. Hence

(88) (∅S ⊙~ (a]λ ⊙~ ∅S)(b)=λ⊇δ, b∈(SaS]

by Theorem 41.

Conversely, assume that *b* ∈ (*S* *aS*] and *δ*⊆*λ*. For any *x* ∈ *S*, if *x* ∈ (*b*] then *x* ∈ (*b*]⊆((*S* *aS*]] = (*S* *aS*]. It follows from Theorem 41 that $\left(\emptyset_{S}\;\tilde{⊙}\;{\left(a\right]}_{\lambda }\;\tilde{⊙}\;\emptyset_{S}\right)(x)=\lambda ⊇\delta ={\left(b\right]}_{\delta }(x)$. If *x* ∉ (*b*], then ${\left(b\right]}_{\delta }(x)=U⊇\left(\emptyset_{S}\;\tilde{⊙}\;{\left(a\right]}_{\lambda }\;\tilde{⊙}\;\emptyset_{S}\right)(x)$. Therefore

(89) $$\begin{array}{c} \left(\emptyset_{S},S\right)\;\tilde{⊙}\;\left({\left(a\right]}_{\lambda },S\right)\;\tilde{⊙}\;\left(\emptyset_{S},S\right)\;\tilde{\subseteq }\;\left({\left(b\right]}_{\delta },S\right). \end{array}$$

This completes the proof.

For any subset *D* of *S* and a proper subset *λ* of *U*, let (*F*_*D*_, *S*) and ((*λF*)_*D*_, *S*) be soft sets over *U* given as follows:

(90) FD:S⟶P(U),x⟼{∅,if  x∈D,U,otherwise,(λF)D:S⟶P(U),x⟼{λ,if  x∈D,U,otherwise,

respectively. Obviously, if *D* = (*a*] then (*λF*)_*D*_ = (*a*]_*λ*_.

**Proposition 45 .**

For any nonempty subsets *D* and *E* of *S* and any proper subset *λ* of *U*, we have the following assertions.

(1) $\left({\left(\lambda F\right)}_{D}\;\tilde{⊙}\;{\left(\lambda F\right)}_{E},S\right)=\left({\left(\lambda F\right)}_{\left(DE\right]},S\right)$.
(2) $\left({\left(\lambda F\right)}_{D}\;\tilde{\cup }\;{\left(\lambda F\right)}_{E},S\right)=\left({\left(\lambda F\right)}_{D\cap E},S\right)$.
(3) $\left({\left(\lambda F\right)}_{D}\;\tilde{\cap }\;{\left(\lambda F\right)}_{E},S\right)=\left({\left(\lambda F\right)}_{D\cup E},S\right)$.
(4) (91) $$\begin{array}{c} \left({\left(\lambda F\right)}_{\left(D\right]},S\right)=\left(\overset{\sim }{\underset{a\in D}{⋂}}{\left(a\right]}_{\lambda },S\right). \end{array}$$

**Proof**

(1) If *x* ∈ (*DE*] then *F*_(*DE*]_(*x*) = *∅* and *x* ≤ *ab* for some *a* ∈ *D* and *b* ∈ *E*. Hence (*a*, *b*) ∈ *A*_*x*_; that is, *A*_*x*_ ≠ *∅*, and thus

(92) ((λF)D ⊙~ (λF)E)(x)  =⋂(y,z)∈Ax{(λF)D(y)∪(λF)E(z)}  ⊆(λF)D(a)∪(λF)E(b)  =λ∪λ=λ.

Since (*λF*)_*D*_(*y*)⊇*λ* and (*λF*)_*E*_(*z*)⊇*λ* for all *y*, *z* ∈ *S*, we get $\left({\left(\lambda F\right)}_{D}\;\tilde{⊙}\;{\left(\lambda F\right)}_{E}\right)(x)⊇\lambda$. Therefore $\left({\left(\lambda F\right)}_{D}\tilde{⊙}{\left(\lambda F\right)}_{E}\right)(x)=\lambda ={\left(\lambda F\right)}_{\left(DE\right]}(x)$.

If *x* ∉ (*DE*] then (*λF*)_(*DE*]_(*x*) = *U*. For the case *A*_*x*_ = *∅*, we have

(93) $$\begin{array}{c} \left({\left(\lambda F\right)}_{D}\;\tilde{⊙}\;{\left(\lambda F\right)}_{E}\right)\left(x\right)=U={\left(\lambda F\right)}_{\left(DE\right]}\left(x\right). \end{array}$$

The case *A*_*x*_ ≠ *∅* implies that *x* ≤ *yz* for all (*y*, *z*) ∈ *A*_*x*_. If *y* ∈ *D* and *z* ∈ *E*, then *yz* ∈ *DE* and so *x* ∈ (*DE*]. This is impossible, and thus *y* ∉ *D* or *z* ∉ *E*. If *y* ∉ *D*, then (*λF*)_*D*_(*y*) = *U* and thus (*λF*)_*D*_(*y*)∪(*λF*)_*E*_(*z*) = *U*. Similarly, if *z* ∉ *E* then (*λF*)_*D*_(*y*)∪(*λF*)_*E*_(*z*) = *U*. Therefore

(94) ((λF)D ⊙~ (λF)E)(x)  =⋂(y,z)∈Ax{(λF)D(y)∪(λF)E(z)}=U.

Consequently, (1) is true.

(2) Let *x* ∈ *S*. If *x* ∈ *D*∩*E*, then *x* ∈ *D* and *x* ∈ *E*, and so

(95) (λF)D∩E(x)=λ=λ∪λ=(λF)D(x)∪(λF)E(x)=((λF)D ∪~ (λF)E)(x).

Assume that *x* ∉ *D*∩*E*. Then (*λF*)_*D*∩*E*_(*x*) = *U*. If *x* ∉ *D*, then (*λF*)_*D*_(*x*) = *U* and so

(96) ((λF)D ∪~ (λF)E)(x) =(λF)D(x)∪(λF)E(x) =U=(λF)D∩E(x).

Similarly, if *x* ∉ *E* then $\left({\left(\lambda F\right)}_{D}\;\tilde{\cup }\;{\left(\lambda F\right)}_{E}\right)(x)={\left(\lambda F\right)}_{D\cap E}(x)$. Therefore

(97) $$\begin{array}{c} \left({\left(\lambda F\right)}_{D}\;\tilde{\cup }\;{\left(\lambda F\right)}_{E},S\right)=\left({\left(\lambda F\right)}_{D\cap E},S\right). \end{array}$$

(3) Let *x* ∈ *S*. If *x* ∈ *D* ∪ *E*, then *x* ∈ *D* or *x* ∈ *E*, which implies that (*λF*)_*D*_(*x*) = *λ* or (*λF*)_*E*_(*x*) = *λ*. Hence

(98) (λF)D∪E(x)=λ=λ∩λ=(λF)D(x)∩(λF)E(x)=((λF)D ∩~ (λF)E)(x).

Suppose that *x* ∉ *D* ∪ *E*. Then *x* ∉ *D* and *x* ∉ *E*. It follows that

(99) ((λF)D ∩~ (λF)E)(x)  =(λF)D(x)∩(λF)E(x)  =U=(λF)D∪E(x).

Therefore $\left({\left(\lambda F\right)}_{D}\;\tilde{\cap }\;{\left(\lambda F\right)}_{E},S\right)=\left({\left(\lambda F\right)}_{D\cup E},S\right)$.

(4) Let *x* ∈ *S*. If *x* ∈ (*D*], then *x* ≤ *b* for some *b* ∈ *D*. Hence

(100) $$\begin{array}{c} \left(\overset{\sim }{\underset{a\in D}{⋂}}{\left(a\right]}_{\lambda }\right)\left(x\right)=\underset{a\in D}{⋂}{\left(a\right]}_{\lambda }\left(x\right)\subseteq b_{\lambda }\left(x\right)=\lambda . \end{array}$$

Note that (*a*]_*λ*_(*x*)⊇*λ* for any critical soft point (*a*]_*λ*_ over *U*. Thus

(101) $$\begin{array}{c} \left(\overset{\sim }{\underset{a\in D}{⋂}}{\left(a\right]}_{\lambda }\right)\left(x\right)=\underset{a\in D}{⋂}{\left(a\right]}_{\lambda }\left(x\right)⊇\lambda . \end{array}$$

Conditions (100) and (101) induce

(102) $$\begin{array}{c} \left(\overset{\sim }{\underset{a\in D}{⋂}}{\left(a\right]}_{\lambda }\right)\left(x\right)=\lambda ={\left(\lambda F\right)}_{\left(D\right]}\left(x\right). \end{array}$$

If *x* ∉ (*D*], then

- (*λF*)_(*D*]_(*x*) = *U* and
- *x* ∉ (*a*] for all *a* ∈ *D*, and so (*a*]_*λ*_(*x*) = *U* for all *a* ∈ *D*.

It follows that

(103) $$\begin{array}{c} \left(\overset{\sim }{\underset{a\in D}{⋂}}{\left(a\right]}_{\lambda }\right)\left(x\right)=\underset{a\in D}{⋂}{\left(a\right]}_{\lambda }\left(x\right)=U={\left(\lambda F\right)}_{\left(D\right]}\left(x\right). \end{array}$$

Therefore

(104) $$\begin{array}{c} \left({\left(\lambda F\right)}_{\left(D\right]},S\right)=\left(\overset{\sim }{\underset{a\in D}{⋂}}{\left(a\right]}_{\lambda },S\right). \end{array}$$

**Theorem 46 .**

If *D* is a left ideal of *S*, then ((*λF*)_*D*_, *S*) is a union-soft *l*-ideal over *U*.

**Proof**

Suppose that *D* is a left ideal of *S*. Let *x*, *y* ∈ *S*. If *y* ∈ *D*, then *xy* ∈ *D* and so (*λF*)_*D*_(*xy*) = *λ* = (*λF*)_*D*_(*y*). If *y* ∉ *D*, then (*λF*)_*D*_(*y*) = *U*⊇(*λF*)_*D*_(*xy*). Assume that *x* ≤ *y*. If *y* ∈ *D*, then *x* ∈ *D* and thus (*λF*)_*D*_(*x*) = *λ* = (*λF*)_*D*_(*y*). If *y* ∉ *D*, then (*λF*)_*D*_(*y*) = *U*⊇(*λF*)_*D*_(*x*). Therefore ((*λF*)_*D*_, *S*) is a union-soft *l*-ideal over *U*.

In the same way, we can verify the following result.

**Theorem 47 .**

If *D* is a right ideal of *S*, then ((*λF*)_*D*_, *S*) is a union-soft *r*-ideal over *U*.

## Figures and Tables

**Figure 1:**